# Facing the Global Challenges of Access to Cancer Medication

**DOI:** 10.1200/JGO.17.00205

**Published:** 2018-03-28

**Authors:** Abdul Raman Jazieh, Abdulaziz H. Al-Saggabi, Mark McClung, Robert Carlson, Lowell E. Schnipper, Alexandru Eniu, Barri Blauvelt, Yousuf Zafar, David Kerr

**Affiliations:** **Abdul Raman Jazieh** and **Abdulaziz H. Al-Saggabi**, National Guard Health Affairs, Riyadh, Saudi Arabia; **Mark McClung**, Amgen Oncology, Thousand Oaks, CA; **Robert Carlson**, National Comprehensive Cancer Network, Fort Washington, PA; **Lowell E. Schnipper**, Beth Israel Deaconess Medical Center; **Barri Blauvelt**, University of Massachusetts, Boston, MA; **Alexandru Eniu**, Cancer Institute “Ion Chiricuta”, Cluj-Napoca, Romania; **Yousuf Zafar**, Duke Cancer Institute, Durham, NC; and **David Kerr**, Oxford University, Oxford, United Kingdom.

## INTRODUCTION

Recent articles and an editorial^1-3^ published in *Journal of Global Oncology* have broached the question of affordability of modern anticancer medication and suggested that “the financial challenge presented by the rising cost of care will create a barrier to its delivery.” Booth and Del Paggio,^2^ as well as Del Paggio et al,^4^ applied both the European Society for Medical Oncology (ESMO) Magnitude of Clinical Benefit Scale and the ASCO Value Framework and concluded that many of these recently approved agents offer only marginal value.

Access to cancer treatment is a major challenge that is encountered in all countries in different forms, such as lack of insurance coverage, copayment, and unavailability of medication in governmental hospitals in countries with a national health coverage system because of lack of approval by Health Technology Assessment (HTA) bodies that is compounded by the inability of the patient to cover the cost by out-of-pocket payment. It is obvious that these factors will be enormously amplified in low- and middle-income countries.^5^

**Fig 1:**
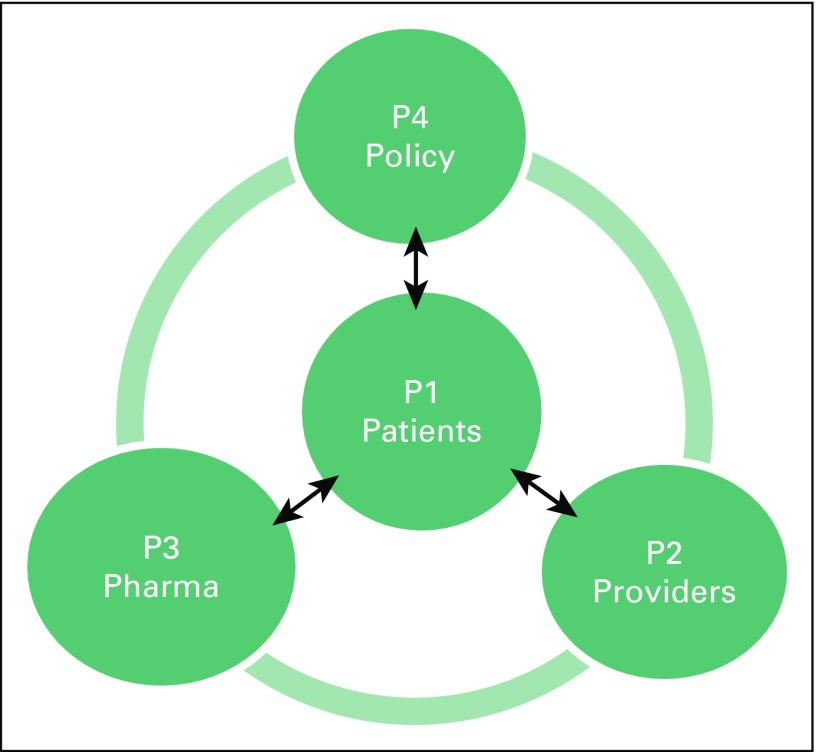
The 4P model: stakeholders in access to medications.

Solutions will only be found by constructing a forum where the relevant players (patients, providers, payers, policy makers, and the pharmaceutical industry) might come together to reflect on a value-based approach to cancer care (Fig 1 and Table 1). The concept of value-driven cancer care is somewhat controversial, because the semantics might suggest that value in this context means lower-quality care. A patient suggested to one of us that, to them, value is more associated with K-Mart than Harrods, and posed the question: “Where would one prefer to shop?”

**Table 1:**
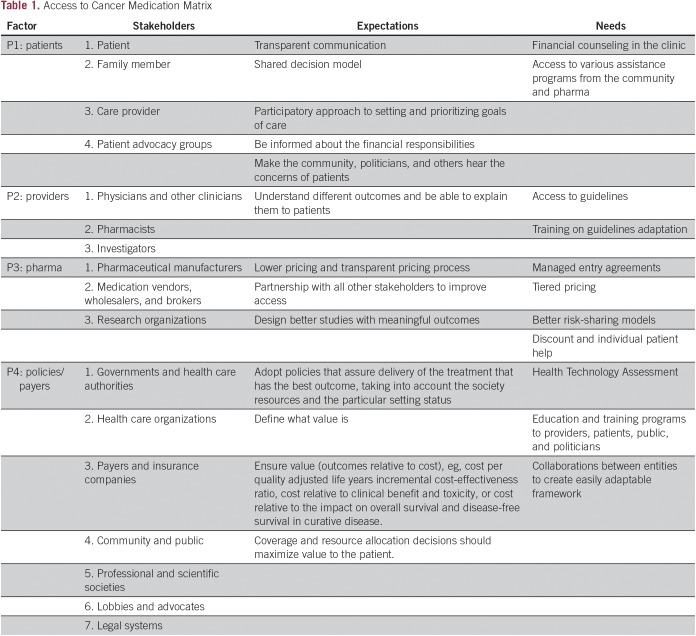
Access to Cancer Medication Matrix

We use Michael Porter’s definition of value: “Value in any field must be defined around the customer, not the supplier. Value must also be measured by outputs, not inputs. Hence it is patient health results that matter, not the volume of services delivered. But results are achieved at some cost. Therefore, the proper objective is to deliver good patient health outcomes relative to the total cost (inputs). Efficiency, then, is subsumed in the concept of value."^6^ Using this definition and relying on all major stakeholders, we suggest potential value-based solutions to overcoming the global challenge of high-quality cancer care delivery.^6^

## P1: PERSONAL AND POPULATION APPROACHES TO THE PATIENT

The main focus of discussion with the patient should be about setting goals of care—taking into account the treatment outcome and the patient’s preferences, needs, and abilities to comply with treatment demands, toxicities both medical and financial, as well as inconvenience. Rather than sweeping cost under the carpet, it is better to deal directly with patient concerns systematically. At extremes, patients may elect not to undergo treatment with marginal benefits to save money for their family, whereas others may go as far as cutting down on all expenditures—even purchasing food—to meet medical bills.^7^ The diagnosis of cancer remains the most common cause of medical bankruptcy, and patients with cancer who declare bankruptcy are at significantly greater risk for mortality.^8^

In striving for value-based care, shared decision making is at the heart of better care of patients with cancer and outcomes, especially in lower-resourced countries. Shared decision making (SDM)^9^ may provide a solution to these intensified challenges faced by lower-resourced countries. SDM has been defined as “an approach where clinicians and patients share the best available evidence when faced with the task of making decisions, and where patients are supported to consider options, to achieve informed preferences.”^9^^(p1361)^

In SDM, the patient is the expert on his/her wants, needs, and values (and personal resources), whereas the clinician is the expert on medical choices and options. What most clinicians must be prepared to include in this is the knowledge of what may be provided by government or private health plans to that patient versus what may have to be paid for directly by the patient. The SDM process involves three Ds: discussion, deliberation, and decision.

Easy access to medical (mis)information enables patients to search available treatment options, so the possibility of ignoring or refusing to acknowledge and discuss the latest treatment advances is neither probable nor just. Physicians should be prepared to discuss options with their patients and explain to them the pros and cons of any particular therapy, using absolute survival benefits rather than proportional reductions, and offset this against cost. Of note, the physician is not alone in finding means for cost reduction. In this effort, social workers, financial counselors, and pharmacists should play an integral role. The value discussion with patients will be assisted by adapting the value assessment models provided by ASCO, ESMO, and National Comprehensive Cancer Network (NCCN) into a software-based tool that is simple and user friendly. At a personal level, the pharmaceutical industry may be able to provide a means-tested wider access for poorer patients.^10-12^

At a population level, perhaps it is time for advocacy by patients—past, present, and future. Taking a page from the AIDS activists, the passion of patients will contribute to a more concerted effort to influence policy makers and industry to develop an improved model to widen anticancer drug access.

## P2: PROVIDERS

Cancer care providers (physicians, nonphysician clinical staff such as nurse practitioners or physician assistants, pharmacists) must be the patient’s primary advocate. They have a central role in facilitating access to cancer therapeutics and will be prescribing and delivering the treatment to the patient.

Physicians must be able to weigh the evidence for a specific indication and prioritize equivalent options. Access to up-to-date guidelines from ASCO, ESMO, and NCCN, as well as the ability to appraise the evidence, is critically important. ASCO^10^ and ESMO^11^ developed value assessment frameworks and NCCN created two new tools to help practitioners adapt their guidelines to their local setting.^12^ Their guidelines can be viewed within the following framework: basic, core, and enhanced resources.

### Basic Resources

Basic resources include essential services needed to provide the basic minimal standard of care.

### Core Resources

Core resources include those provided in the basic resources framework plus additional services that provide major improvements in disease outcomes (eg, survival) and that are not cost prohibitive.

### Enhanced Resources

Enhanced resources include those provided in the core resources framework and additional services that provide lesser improvements in disease outcomes and/or services that provide major improvements in disease outcomes but are cost prohibitive in lower-resource settings.

In addition, the NCCN evidence blocks^12^ are a simple visual representation of five components of value including efficacy, safety, strength of evidence, consistency of evidence, and affordability, which can be used by the practitioner to make a transparent presentation of treatment options and deliver the protocol that offers greatest value to the patient.

Because there are several frameworks—each measuring clinical benefit and toxicity—an essential next step is to attempt to reconcile differences in how each measures these variables. A uniform set of standards, albeit challenging to achieve, would enhance the value frameworks and promote standards to which the biotech and pharmaceutical industries can aspire.

## P3: PHARMACEUTICAL INDUSTRY

The pharmaceutical industry represents an essential resource and is to be credited with developing anticancer therapies that, in a few diseases, have had profound impact. However, many new products introduced to the market have only marginal benefits yet are costly.

The current debate about prescription drug pricing is complex and centers on determining the most appropriate basis for calculating how payers (including patients, government agencies, employers, and health plans) should pay pharmaceutical companies, pharmacies, and other providers for dispensing prescription drugs and providing pharmaceutical services. The drivers underpinning this debate include the following:

Growth of health care as a percentage of gross domestic product.
Payer demands for price transparency.
Increasing cost sharing by patients.
The belief by many stakeholders that prescription drug prices and price increases should be moderated.
Increase in specialty pharmaceuticals on the market and their increasingly high cost per course.
Undisclosed prescription drug rebates and discounts, which may differ by type of purchaser.

Several critical factors are believed to contribute to the rapidly rising cost of anticancer drugs and must be addressed.

1. There are huge costs in bringing a drug from the bench to the bedside. Cumbersome regulations, large human trials because of the lack of effective biomarkers predicting response, and regulations governing clinical research are all factors. It is essential to reduce the regulatory burden, as well as to identify alternative trial designs to assure smaller trials that would reduce the cost of drug development.
2. The costs of new agents bear no relationship to the clinical impact attributable to the drug.^13^ The value frameworks can provide guidance regarding the magnitude of clinical benefit provided by a new drug, and that fact should be used in negotiating a fair price.

The pharmaceutical industry includes not only the inventors and manufacturers, but a whole chain of vendors, brokers, distributors, and wholesalers, each of whom will contribute to total drug costs. The expectation from pharmaceutical companies is to have transparent and clear pricing processes that allow them to explain to stakeholders what is included in the cost of development of new medications. At a macro level, we do need to develop more collaborative, shared risk models for clinical research to reduce the increasingly prohibitive costs of taking a new drug to market. Any modification of the system must encourage innovation by assuring substantial rewards for major breakthroughs, and implicitly, less of a reward for a new product with a minimal impact.

The following are practical steps that can be taken by the industry to increase drug availability^11^:

Managed entry agreements can take different forms, including price-volume agreements, outcome guarantees, coverage with evidence development, and disease management programs. A variety of names have been used to describe these schemes (eg, risk-sharing agreements, performance-based agreements, patient access schemes, and so on).
Tiered pricing will be an option to account for the widely variant economic status of different countries, and risk-sharing models would help to facilitate the delivery of medications to poorer patients.
Individual patient assistance programs should be available across various systems and across boundaries. These should be announced and disseminated to all stakeholders because, on many occasions, providers and patients may not know about their availability.

## P4: POLICY/PAYERS

Many stakeholders are involved in policy development and are often isolated from one another (governments and health care authorities including HTA centers, payers and insurance companies, professional and scientific bodies, lobby and advocacy groups, and the legal systems).

Internationally, there are vastly different model systems for health care, including cancer care that ranges from universal coverage to partial coverage to no coverage at all. The variables that dictate these national policies include political, economic, and social drivers. However, the general, unifying philosophy is that most governments and associated actors would seek to find a cost-effective model for the delivery of basic cancer care within their financial means. The UK’s National Cancer Plan is a beacon of such a strategy,^14^ whereby the government created and funded a wide-ranging, multidisciplinary action plan to improve the nation’s dismal survival figures, with some success.^14^

Most developed countries with national health care coverage use a fairly uniform HTA model to decide whether a specific medication will be provided to their patients (such as the UK’s National Institute for Clinical Excellence).^15^

Other nations cover cancer care for their patients but do not have a centralized HTA process. In such cases, the decisions devolve to their tertiary institutions (eg, as in Saudi Arabia) or to a mélange of private insurance companies and the government (as is the case in the United States). Alas, there are many low- and middle-income countries that have no cancer plan and de minimis cancer care systems. For those nations, the WHO has compiled a list of essential cancer medicines^10^ that serve as an invaluable guide to those clinicians and advocates who are making a case for basic cancer care in low-income countries. The problem in this scenario is that some low- and middle-income countries do not have the financial capability to provide even those on the essential medicines list for their citizens.

As the nations of the world struggle to find the optimal path toward universal access to affordable cancer care, a key element will be an agreed-upon set of metrics to evaluate cancer treatments and to quantify the clinical value they provide. Governments, insurance companies, and other payers can mandate tumor-specific patient pathways and clinical guidelines, an area in which professional societies and other representative bodies can make internationally recognized contributions (eg, ASCO, ESMO, and NCCN). One would expect that economic forces would then come into play and lead to adjustment in price in accordance with a product’s utility.

Health policy may be driven by ideology (left-wing *v* right-wing philosophy), evidence, (when data supporting a specific health intervention are so compelling that its introduction is a no-brainer), and emotion (eg, how often have we, the public, and the body politic been moved by a direct appeal through the media for access to a life-saving drug by a child, a mother, a brother?). Individuals might choose to ally themselves as policy advisers to particular political parties; physician groups and professional societies can play a role in providing high-quality data and educating politicians and the public about the real benefits of any novel therapy; activists may choose to unite behind an individual patient’s story to get a wider message into the media.

## PAYERS

Although often caught between the pharmaceutical industry’s high drug prices and the demand driven by patients and providers, the insurance industry still has a role to play in the delivery of high-value care. Its role can be mostly summarized into two broad categories: 1) championing the use of high-value anticancer therapy; and 2) limiting patient cost-sharing for those same therapies.

First, payers—especially those in the United States—can identify high-value interventions and encourage their use, while simultaneously limiting the use of low-value interventions. If payers were not mandated to cover all available cancer therapies, they would have more leverage with the pharmaceutical industry to reduce prices, with the potential for noncoverage.^16^ Another powerful means for payers to promote value in cancer is in the form of value-based insurance design, wherein high-value interventions are covered with little cost-sharing, while low-value interventions are either not covered at all or are covered with high rates of cost-sharing with the intent to limit use.

Value-based insurance design is also useful in reducing costs to patients; this is a second important role that payers can play in promoting value. Numerous studies have suggested a strong association between high out-of-pocket costs for anticancer therapy and nonadherence to that therapy.^17-19^ Even copayments as low as approximately $50 per month might induce nonadherence to potentially life-prolonging therapy, such as imatinib for chronic myelogenous leukemia.^17^ Payers should limit or remove altogether cost-sharing in the form of copayments for high-value interventions; nonadherence because of cost not only worsens care but will ultimately increase costs for payers and society.

## CONCLUSION

It is clear that a more coordinated, global effort needs to be made to bring together the necessary stakeholders to provide a range of solutions that can be adapted to local circumstances (Table 1). Each country or setting should work on defining the relevant factors for its own system and select the best approach to enhance access to cancer therapy in a meaningful way. Although creating policy and process are paramount, assuring a competent cancer care workforce skilled in the knowledge of clinical guidelines, cost-effective treatment options as derived from contemporary value assessment tools, and expertise in how to communicate effectively with patients to explain their choices in detail are critical first steps. 
